# Post-traumatic Growth in the First COVID Outbreak in Hong Kong

**DOI:** 10.3389/fpsyg.2021.675132

**Published:** 2021-09-16

**Authors:** Bobo Hi Po Lau, Cecilia Lai Wan Chan, Siu Man Ng

**Affiliations:** ^1^Department of Counselling and Psychology, Hong Kong Shue Yan University, North Point, Hong Kong, SAR China; ^2^Wan Chow Yuk Fan Centre for Interdisciplinary Evidence-Based Practice & Research, Hong Kong Shue Yan University, Hong Kong, Hong Kong, SAR China; ^3^Centre on Behavioral Health, University of Hong Kong, Hong Kong, Hong Kong, SAR China; ^4^Department of Social Work and Social Administration, University of Hong Kong, Hong Kong, Hong Kong, SAR China

**Keywords:** COVID-19, post-traumatic growth, sense of coherence, post-traumatic stress, Hong Kong, Chinese, perceived severity, coping

## Abstract

Post-traumatic growth (PTG) emerges from highly stressful situations. The coronavirus (COVID) pandemic may qualify as one. This study investigated the PTG among Hong Kong citizens during the first outbreak in spring 2020, shortly after a large-scale social movement subsided. A longitudinal online survey was launched during the peak (Time 1) and the palliation (Time 2) of the outbreak. Among the 327 participants who completed both waves, 28.4% exhibited probable post-traumatic stress disorder (PTSD) in Time 1, while 18.0% reported significant PTG in at least one domain in Time 2. The interaction between the sense of coherence (SOC) and post-traumatic stress mediated the relationship between Time 1 perceived outbreak severity and Time 2 PTG, such that PTG was more likely among participants with higher post-traumatic stress and SOC. PTG was also associated with a weaker contingency between Time 1 and Time 2 perceived outbreak severity. Echoing the transformational model, our findings show that both experienced stress and coping resources are essential for PTG to emerge. We also demonstrated how PTG might lead to more flexible risk perceptions according to the development of the outbreak.

## Introduction

The coronavirus (COVID) pandemic has hit hard globally, leading to more than 113 million cases and 2.5 million deaths by March 2021 (Dong et al., 2020). The novelty of the viral pandemic and its pervasive impact on personal lives, and the economy have resulted in challenges of unprecedented scale to the global community. Elevated percentages of post-traumatic stress (PTS) have been documented in studies across continents, e.g., U.S., 31.8% (Liu et al., 2020), the U.K., 16.8% (Shevlin et al., 2020), Spain, 15.8% (González-Sanguino et al., 2020), and Lebanon, 33.0% (Fawaz and Samaha, 2020). McKinsey and Company (2020) have estimated that in the U.S. alone, additional 35 million individuals with mental health needs will emerge due to the direct losses from COVID (e.g., loss of health and bereavement) and the indirect impacts of the pandemic on the healthcare system, the economy, and the society.

The pandemic experience of Hong Kong has been distinctive as it was the epicenter of the Severe Acute Respiratory Syndrome (SARS) in 2003 (a relatively recent COVID epidemic) and has endured months of social unrest in 2019–2020, shortly before the first local outbreak. The SARS epidemic has resulted in 1,755 cases and 299 deaths Leung et al. (2009). Like COVID, scientists have the minimal idea on the means of transmission and impacts of SARS at the beginning of the epidemic. The epidemic has been costly, both economically and socially. Between 1997 and 2018, the unemployment rate of the city was the highest in 2003 (8.5%; Hou et al., 2021b), so was the suicide rate (18.8 per 100,000; HKJC Centre for Suicide Research Prevention, 2020). A public mental health crisis was resulted with 13.3–18.0% respondents of a population-based telephone survey reporting marked PTS (Lau et al., 2005).

In mid-2019, an intense social movement sparked off by the introduction of the controversial Extradition Law Amendment Bill (ELAB) has generated another public mental health crisis (Hou and Hall, 2019). The social movement has quickly escalated from peaceful mass demonstrations into widespread and intense clashes between the protesters and the police (Ting, 2020). Ni et al. (2020) found a prevalence of 11.2% for depression and 12.8% for probable post-traumatic stress disorder (PTSD) during the heights of the movement in late 2019, compared to just 1.9% for depression before the 2014 Umbrella Movement (UM) and 4.9% for probable PTSD shortly after the UM. Hou et al. (2021a) further reported a prevalence of 9.1% for suicidal ideation.

The emergence of the first COVID case in Hong Kong on January 23, 2020 has alarmed the city for another tough test. Although the SARS experience may have inoculated Hong Kong citizens with high vigilance toward the COVID outbreak (Kwok et al., 2020; Lau et al., 2020a), the “double-hit” from the novel viral outbreak and the social unrest have generated substantial mental distress among the citizens, especially under the severely shattered trust to the public authority (Ni et al., 2020) and a highly divisive social fabric (Wong, 2020). Although, most protests had subsided at the beginning of 2020 when COVID arrived. Hou et al. (2021a) reported high rates of probable depression and anxiety (21.8–33.9%) among respondents who experienced substantial stress from both the pandemic and the sequelae of the unrest, and these rates were higher compared to those of respondents who reported stressing out for either the pandemic or the unrest only.

Despite the psychopathological consequences of highly stressful events, increasingly researchers have been inquiring whether and how people grow through these challenges. PTG, defined as the positive psychological changes upon having to terms with highly stressful events (Tedeschi and Calhoun, 2004), has been witnessed in various contexts, ranging from earthquakes (Nakagawa et al., 2016; First et al., 2018) to attacks (Park et al., 2012). Substantial positive mental-health impacts have also been reported with the SARS epidemic locally (Lau et al., 2006), including greater perceived support from the loved ones (22.0–39.1%), greater attention paid to one's mental health (65.8–65.9%), and adoption of healthier lifestyles (35–40%). The affective blunder of the stressful event, rather than the event alone, motivates constructive cognitive processing. In turn, such cognitive processing may lead to a change in perspectives about oneself, the event, and the world around (Kashdan and Kane, 2011; Park et al., 2012; First et al., 2018). In other words, the struggle to make sense of and attain meaning from the distressing situation is where the growth emerges. Under the unique backdrop of the “double-hit” from the COVID pandemic and the sequelae of the social unrest in Hong Kong, this study was conducted to investigate the development of PTG after the first local outbreak in Spring 2020.

Some people tend to possess a higher tendency to engage in the constructive deliberation behind PTG than others. According to the salutogenesis theory (Antonovsky, 1979), sense of coherence (SOC) functions as a global orientation to cope with one's stressors with the general resistance resources at one's disposal (e.g., material, knowledge, social networks, personality, faith, and religion, etc.). SOC encompasses the cognitive, behavioral, and motivational impetuses of one's coping efforts, such as comprehensibility (i.e., perceiving the nature of the stressor as predictable, orderly, and explicit), manageability (i.e., perceiving the stressor as one that can be handled adequately by one's resources), and meaningfulness (i.e., perceiving coping efforts as worthwhile of investing energy and commitment rather than just a burden). Previous studies have shown that people with higher SOC tend to cope with highly stressful events with more positive reframing, direct action, and social support, and in turn derive greater PTG (López et al., 2015; Ragger et al., 2019; Schäfer et al., 2019).

**Figure 1:**
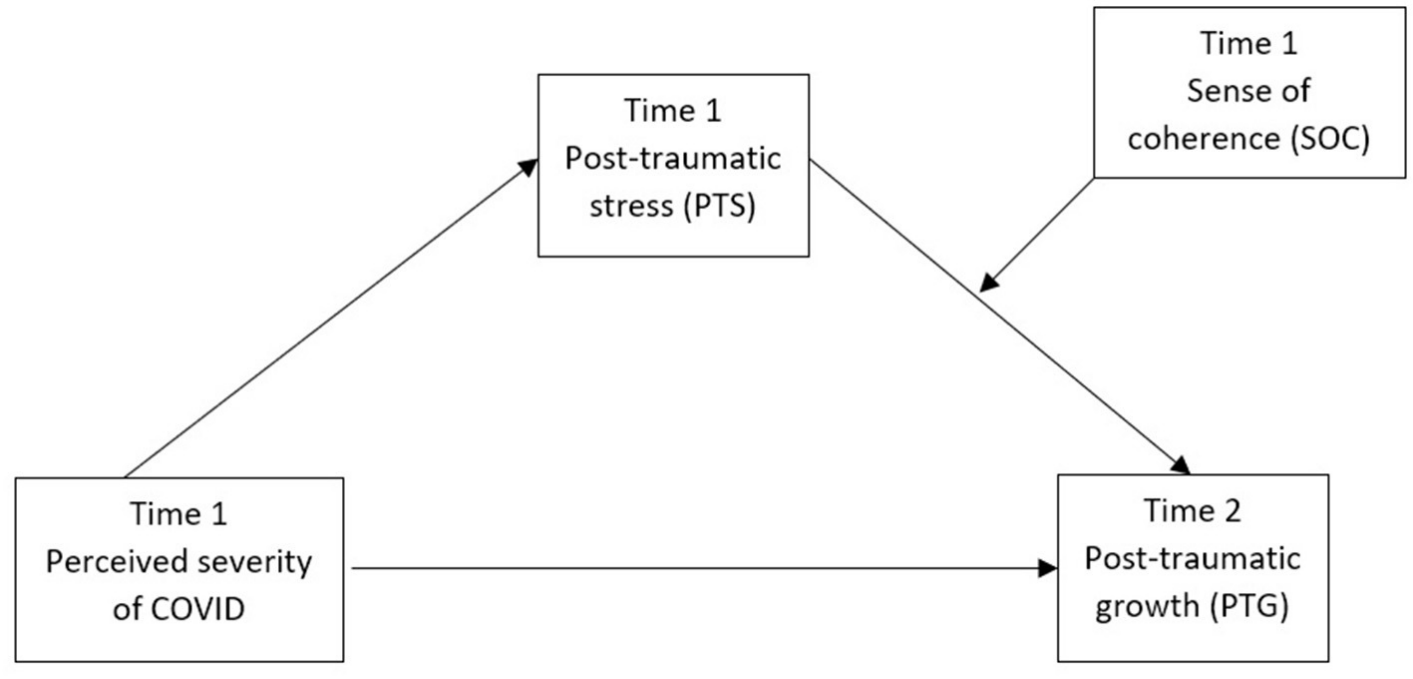
Schematic diagram of the moderated mediation model on the emergence of post-traumatic growth at Time 2 from perceived severity of COVID at Time 1 through a combination of high post-traumatic stress and high sense of coherence.

Based on the transformational model (Tedeschi and Calhoun, 2004), one has to experience the event as psychologically “seismic,” meaning that the experience has to be distressing enough, for PTG to occur. PTG is, however, not a necessary outcome of a highly distressing event. People need to “use” their coping resources for constructive deliberation to generate refreshed and resilient perspectives, and SOC is a good candidate for such coping resources (Schäfer et al., 2019). Hence, we tested a moderated mediation model explaining the emergence of PTG from a longitudinal survey conducted at the peak (Time 1) and the trough (Time 2) of the first local outbreak in Spring 2020. The following hypothesis was put forward:

Hypothesis 1: The interaction between SOC and PTS at Time 1 mediates the relationship between perceived severity of COVID at Time 1 and PTG at Time 2, such that participants with higher SOC and PTS will experience a stronger positive relationship between perceived severity and PTG (Figure 1).

Furthermore, PTG is analogous to quake-proof buildings built after a geoseismic event. That is, it fosters transcendence beyond the pre-trauma status and builds a psychological infrastructure with more resilient perspectives on oneself, one's interpersonal circle and spirituality, and in turn affords one with greater resistance against further psychological harms (Tedeschi and Calhoun, 2004). Hence, we postulated that PTG will be conducive to the flexibility of pandemic severity judgments, resulting in the participants being less cognitively aroused by COVID when the outbreak has been palliated. Such flexibility could be indicated by a weaker association between Time 1 and 2 perceived severity ratings of COVID.

Hypothesis 2: The relationship between Time 1 and 2 perceived severity of COVID will be weaker among participants with higher PTG at Time 2.

## Methods

### Context

This research has been launched right after the WHO declared COVID a pandemic (Time 1: March 12, 2020 to April 8, 2020). At that time, Hong Kong was experiencing its first major wave of a local outbreak, with an average of more than 30 cases per day and each additional 100 cases took <3 days to accrue. Several infection clusters from karaoke lounges, hotpot restaurants, gyms, and bars have been identified. Despite the suspension of schools and most public services since late-January, 2020, the government had first introduced public health orders, such as gathering bans, mandatory closure of premises and businesses, and catering restrictions in restaurants (reduced service capacity and mandatory inter-table distance) in late March. In late April and May, the outbreak subsided to an average of only one case per day, and we conducted the Time 2 (April 24, 2020–May 12, 2020) data collection.

### Recruitment and Participants

Eligible participants were Hong Kong Chinese residents aged 18 or above. Individuals who cannot read traditional Chinese or have no access to the internet were excluded. Conducted as a swift response to the highly versatile situation, participants were recruited through snowballing *via* social media (e.g., Facebook pages, WhatsApp groups, and Telegram channels) and email lists. Participants were rewarded an HKD﹩50-supermarket coupon after completing each survey.

### Instruments

#### Perceived Severity

Perceived severity of the COVID outbreak was measured by two items in Time 1 – “On a scale of 1 (not at all) to 10 (extremely severe), how severe do you think the current outbreak is” and “compared to SARS in 2003, how severe do you think the current outbreak is on a scale from 1 (much less severe than SARS) to 5 (the same), and 10 (much more severe than SARS.” The two items achieved good reliability (Cronbach alpha = 0.75). In Wave 2, only the first item was adopted, as the local and international community have arrived at a consensus that COVID is distinct from SARS.

#### SOC

The 13-item SOC (SOC-13; Antonovsky, 1987) was used to measure comprehensibility, manageability, and meaningfulness under the salutogenesis model. Participants answered on 7-point scales, and the scale score was obtained from summing up the responses across the items. The Cronbach alpha was 0.85, indicating good reliability.

#### PTS

The 22-item Impact of Event Scale—Revised (IES-R; Weiss, 2007) was adopted. The scale taps into three major areas of PTS: hyperarousal, intrusion, and avoidance. The items were re-worded to specifically measure the impacts of the current COVID pandemic (Ng, 2020). Participants were asked to refer to their experience in the past 7 days and answered on a 5-point scale running from 0 (not at all) to 4 (extremely). The scale score was derived from summing up the items, and a score above 32 indicates probable PTSD (Creamer et al., 2002). The scale showed excellent reliability (alpha = 0.95).

#### PTG

The 21-item Post-traumatic Growth Inventory (PTGI; Tedeschi and Calhoun, 1996) was adopted. Participants were instructed to answer based on their experience with the pandemic. According to Ho et al. (2004), the Chinese version contains four dimensions, i.e., Self (items 8, 9, 10, 11, 13, 19, and 21), Interpersonal (items 6, 7, and 12), Spiritual (items 4, 5, and 16), and Life orientation (items 1 and 2). Participants answered on a 6-point scale running from 0 (none) to 5 (very much). Responses on the items were averaged to form the scale score, and the attainment of substantial PTG was indicated by an average score of 4 (Ng et al., 2020). The Cronbach alphas of the subscales ranged from 0.51 (Spiritual) to 0.87 (Self), while that of the full scale was 0.94, indicating adequate reliability.

Demographic characteristics, perceived severity, SOC, and PTS were measured at Time 1, while PTG and perceived severity were measured at Time 2.

### Procedure

The study was conducted through online surveys. Participants who completed Time 1 in March were contacted for Time 2 in late April; while those who completed Time 1 in early April were contacted for Time 2 in early May. Hence, there was about a 1-month gap between the two sets of data. The data across time points were matched by phone numbers of participants or email addresses of their choices. All participants provided their informed consent online before beginning the questionnaire. The study was approved by the Human Research Ethics Committee of University of Hong Kong (EA2003003).

### Statistical Analysis

Demographic characteristics related to PTG were explored with unadjusted logistic regressions, whereas the associations among the key variables were investigated with bivariate Pearson's correlations. Moderated mediation models with perceived severity as the predictor (X), PTS as the mediator (M), SOC as the moderator (W), and the PTG overall and subscales as the outcome (Y) were tested with the PROCESS macro version 3.3 with 5,000 bootstrapped samples (Hayes, 2018). Considering the conceptual distinctiveness and asynchronicity of growth in PTG domains (Ho et al., 2004; Ng, 2020), an individual model was fitted for each domain and the overall score. In other words, five moderated mediation models were tested. Statistical significance at *p* < 0.05 of a conditional indirect effect was indicated by a pair of 95% CIs that exclude a zero.

In addition, to test the moderating effect of PTG at Time 2 on the relationship between Time 1 and 2 perceived severity, moderation analyses were conducted. Likewise, PROCESS macro version 3.3 with 5,000 bootstrapped samples was used to estimate the moderating effects, and a separate model was fitted for each domain of PTG, in other words, five moderation models were tested.

In both the moderated mediation models and the simple moderation models, the direction of the moderation effects was examined by scrutinizing the effects of the focal predictor at 16th (i.e., one standard deviation below mean), 50th and 84th (i.e., one standard deviation above mean) percentiles of the moderator. Missing values were handled by list-wise deletion. All analyses were conducted with SPSS 25.0.

## Results

Among the 327 participants (Table 1), 71.9% were female, with an average age of 35.0 (SD = 13.5, range = 18–73). The majority of participants (68.5%) were university educated. The median monthly household income was in the range of HKD﹩40,000–49,999 (US﹩5,128–6,410), which is considerably higher than the population average (at HKD﹩27,000/US﹩3,462). About half of the samples were living with a person vulnerable to a serious course of disease in case of a COVID infection (e.g., toddler, children, pregnant woman, elderly, and person with disability or chronic illness). Total 35.2% of participants were working in an occupation self-perceived as high-risk (e.g., healthcare, retail, catering and beverage, sanitary, and jobs that require traveling aboard, etc.). Five participants had been confirmed positive on COVID, and seven had experienced medical quarantine. Twenty participants had a family member or close friend diagnosed with COVID, and 36 participants had a family member or close friend who had experienced medical quarantine. A majority of participants (88.4%) considered the outbreak at Time 1 to be more severe than SARS, while 28.4% of participants scored above 32 on the IES-R and exhibited probable PTSD.

**Table 1:** Post-traumatic growth by demographic characteristics (*N* = 327).

**Demographic characteristics**	***N*** **(%)**	**% of participants with growth in at least 1 domain of PTG**	**95% CI of OR**
**Gender**			
Female	235 (71.9%)	18.7%	0.62–2.25
Male	92 (28.1%)	16.3%	–
**Age in years**			
18–39	207 (63.3%)	16.9%	0.36–1.97
40–59	79 (24.2%)	20.3%	0.41–2.70
60–79	41 (12.5%)	19.5%	–
**Education**			
University or more	224 (68.5%)	21.4%	1.13–4.60^*^
Less than university	103 (31.5%)	10.7%	–
**Monthly family income**			
HKD﹩40,000 or more	170 (52.0%)	25.3%	1.60–5.56^**^
Less than HKD﹩40,000	157 (48.0%)	10.2%	–
**Marital status**			
Married	154 (47.1%)	17.5%	0.53–1.65
Not married	173 (52.9%)	18.5%	–
**Religious affiliation**			
Yes	140 (42.8%)	20.0%	0.72–2.22
No	187 (57.2%)	16.6%	–
**Own chronic health condition**			
Yes	82 (25.1%)	22.0%	0.75–2.61
No	245 (74.9%)	16.7%	–
**Live with a vulnerable person**			
Yes	158 (48.3%)	19.6%	0.70–2.16
No	169 (51.7%)	16.6%	–
**In a high-risk occupation**			
Yes	115 (35.2%)	19.1%	0.62–2.01
No	212 (64.8%)	17.5%	–
**Oneself tested positive on COVID-19^a^**			
Yes	5 (1.6%)	40.0%	0.50–18.39
No	310 (98.4%)	17.7%	–
**Oneself experienced medical quarantine^a^**			
Yes	7 (2.2%)	14.3%	0.09-6.35
No	308 (97.8%)	18.2%	–
**Family/close friend tested positive on COVID-19^a^**			
Yes	20 (6.3%)	45.0%	1.66–10.71^**^
No	295 (93.7%)	16.3%	–
**Family/close friend experienced medical quarantine^a^**			
Yes	36 (11.4%)	33.3%	1.21–5.58^*^
No	279 (88.6%)	16.1%	–

*95% CI of OR, 95% CI of OR; OR, odds ratio; PTG, post-traumatic growth*.

*HKD﹩40,000 = USD﹩5159*.

a *Missing information from 12 participants*.

* *p < 0.05*;

** *p < 0.01*.

Only seven participants (1.8%) attained substantial PTG (i.e., >4.0) by the overall scale score. Percentages of participants attaining substantial PTG on Self, Interpersonal, Spiritual, and Life orientation were 5.5, 8.3, 4.0, and 7.6%, respectively. About one in five participants (18.0%) showed PTG in either Self, Interpersonal, Spiritual or Life orientation, or a combination of domains, with 13.1% reported PTG in only one of these domains, while the ratios tapered off to 2.8, 1.8, and 0.3% for PTG in two, three, and all four domains, respectively.

The results of the unadjusted logistic regressions show that having an above-sample-median monthly household income (HKD﹩40,000), being tertiary educated, and being with a family member or close friend who has experienced medical quarantine or being tested positive on COVID were related to a higher likelihood of PTG (Table 1). Associations with other demographic factors were non-significant.

Table 2 provides the intercorrelations among the key variables. Perceived severity at Time 1 and 2 was moderately positively correlated. PTS and perceived severity at both Time 1 and 2 were unrelated to PTG Overall and subscales, except being positively associated with life orientation and Time 2 perceived severity with spiritual. SOC was positively related to Overall PTG, Self, and Interpersonal. PTS was negatively related to SOC but positively associated with perceived severity at Time 1.

**Table 2:** Descriptive statistics and intercorrelations among key variables (*N* = 327).

**Variables**	**Mean (SD)**	**2**	**3**	**4**	**5**	**6**	**7**	**8**	**9**
1. Perceived severity (Time 1)	7.95 (1.53)	0.33^***^	0.22^***^	−0.02	0.11	0.08	0.04	0.09	0.17^**^
2. Perceived severity (Time 2)	6.50 (2.20)	–	0.06	−0.04	0.10	0.07	0.22	0.15^**^	0.13*
3. Post-traumatic stress	24.13 (13.93)		–	−0.46^***^	0.03	−0.06	0.01	0.07	0.24^***^
4. Sense of coherence	56.24 (13.93)			–	0.17^**^	0.23^***^	0.13^*^	0.08	−0.06
5. Post-traumatic growth—Overall	2.53 (0.82)				–	0.94^***^	0.82^***^	0.79^***^	0.63^***^
6. Post-traumatic growth—Self	2.62 (0.93)					–	0.72^***^	0.68^***^	0.48^***^
7. Post-traumatic growth—Interpersonal	2.67 (0.94)						–	0.56^***^	0.48^***^
8. Post-traumatic growth—Spiritual	2.30 (0.95)							–	0.49^***^
9. Post-traumatic growth—Life orientation	2.54 (0.99)								

*SD, standard deviation*.

* *p <0.05*;

** *p <0.01*;

*** *p <0.001*.

*Post-hoc* power analysis by G^*^Power 3.1.9.2 suggested the sample achieved over 95% power for linear regression with 12 predictors and 20% variance explained. Table 3 shows the results of the five moderated mediation models. Controlling for demographic variables, Time 1 perceived severity was positively related to PTS. In all models, the interaction between SOC and PTS was significantly associated with PTG. Table 4 illustrates the effect of PTS on PTG at different levels of SOC (16th, 50th, and 84th percentile). Higher levels of SOC were related to more positive relationships between PTS and PTG (Table 4). In fact, negative relationships of PTS on Self and Interpersonal were found at low SOC (16th percentile).

**Table 3:** Moderated mediation models on post-traumatic growth and subscales (*N* = 327).

**Predictors**	**Block 1**	**Block 2**				
	**Outcome: PTS B (SE)**	**Outcome: PTG-Overall B (SE)**	**Outcome: PTG-Self B (SE)**	**Outcome: PTG-Interpersonal B (SE)**	**Outcome: PTG-Spiritual B (SE)**	**Outcome: PTG-Life orientation B (SE)**
Gender	3.70 (1.67)^*^	0.25 (0.10)^*^	0.29 (0.11)^**^	0.31 (0.11)^**^	0.20 (0.11)	0.14 (0.12)
Age	−0.97 (0.06)	0.01 (0.00)	0.01 (0.00)^*^	0.00 (0.00)	0.01 (0.00)^**^	−0.00 (0.00)
Education	3.61 (1.79)^*^	0.10 (0.10)	0.05 (0.12)	0.16 (0.12)	0.14 (0.12)	0.03 (0.13)
Income	−0.83 (0.26)^**^	0.02 (0.02)	0.01 (0.02)	0.01 (0.02)	0.02 (0.02)	0.03 (0.02)
Own chronic health condition	1.73 (1.82)	−0.01 (0.10)	0.01 (0.12)	−0.01 (0.12)	0.02 (0.12)	−0.08 (0.13)
Live with a vulnerable person	1.94 (1.50)	0.05 (0.09)	0.04 (0.10)	0.10 (0.10)	0.03 (0.10)	−0.07 (0.11)
In a high-risk occupation	1.99 (1.57)	0.19 (0.09)^*^	0.23 (0.10)^*^	0.07 (0.11)	0.21 (0.10)^*^	0.04 (0.11)
Religious affiliation	1.23 (1.53)	0.07 (0.09)	−0.00 (0.10)	−0.14 (0.10)	0.58 (0.10)^***^	0.05 (0.11)
Perceived severity (Time 1)	1.59 (0.50)^**^	0.06 (0.03)^*^	0.06 (0.03)	0.03 (0.03)	0.05 (0.03)	0.09 (0.04)^**^
Post-traumatic stress		−0.06 (0.02)^***^	−0.06 (0.02)^**^	−0.07 (0.02)^***^	−0.05 (0.02)^**^	−0.04 (0.02)
Sense of coherence		−0.01 (0.01)	−0.01 (0.01)	−0.02 (0.01) ^*^	−0.02 (0.01)^*^	−0.02 (0.01)
Interaction between post-traumatic stress and sense of coherence		0.00 (0.00)^***^	0.00 (0.00)^***^	0.00 (0.00)^***^	0.00 (0.00)^**^	0.00 (0.00)^**^
**Model summary**						
Change in r^2^	0.1052^***^	0.1484^***^	0.1457^***^	0.1130^***^	0.2073^***^	0.1199^***^

*Five moderated mediation models were tested. As Block 1 of all five models was the same, for simplicity's sake, only one set of results was presented for Block 1*.

B, unstandardized coefficient; SE, standard error; PTG, post-traumatic growth.

* *p < 0.05*;

** *p < 0.01*;

*** *p < 0.001*.

**Table 4:** Effects of post-traumatic stress on post-traumatic growth at 16th, 50th, and 84th percentile of sense of coherence (*N* = 327).

	**Effects [B(SE)] of PTS on PTG domains:**				
	**Overall**	**Self**	**Interpersonal**	**Spiritual**	**Life orientation**
16th percentile of SOC	−0.0068 (0.0046)	−0.0108 (0.0053)^*^	−0.0109 (0.0055)^*^	−0.0054 (0.0052)	0.0067 (0.0057)
50th percentile of SOC	0.0056 (0.0036)	0.0011 (0.0041)	0.0042 (0.0042)	0.0059 (0.0040)	0.0175 (0.0044)^***^
84th percentile of SOC	0.0191 (0.0051)^***^	0.0142 (0.0058)^**^	0.0207 (0.0060)^***^	0.0184 (0.0057)^**^	0.0293 (0.0063)^***^

*B, unstandardized coefficient; SE, standard error; PTS, post-traumatic stress; SOC, sense of coherence; PTG, post-traumatic growth*.

* *p < 0.05*;

** *p < 0.01*;

*** *p <0.001*.

Table 5 provides the magnitude of the conditional indirect effects, and all of them were significant. That is, the interaction between PTS and SOC significantly mediated the relationship between Time 1 perceived severity and PTG in all models. In other words, the moderated mediation mechanism was supported. Only under high SOC (84th percentile) PTS mediated the relationship between perceived severity and PTG and subscales, except for Life Orientation where the mediation was significant even with a moderate level of SOC (50th percentile). In other words, perceiving the outbreak as more severe led to greater PTS; in turn, the coupling between high PTS and high SOC resulted in greater PTG (moderate SOC is enough for the case of Life orientation). Complete mediation was found with Self, Interpersonal, and Spiritual where the direct effects became non-significant. Incomplete mediation with the direct effects remaining significant was found with PTG Overall and Life orientation (see Table 3).

**Table 5:** Conditional indirect effects on post-traumatic growth at 16th, 50th, and 84th percentile of SOC and magnitudes of the moderated mediation (*N* = 372).

	**Indirect effects [B(SE)]** ***via*** **PTS from perceived severity on PTG domains:**				
	**Overall**	**Self**	**Interpersonal**	**Spiritual**	**Life orientation**
16th percentile of SOC	−0.0108 (0.0093)	−0.0172 (0.0111)	−0.0173 (0.0114)	−0.0087 (0.0094)	0.0106 (0.0113)
50th percentile of SOC	0.0089 (0.0070)	0.0018 (0.0070)	0.0067 (0.0076)	0.0094 (0.0082)	0.0278 (0.0117)^*^
84th percentile of SOC	0.0303 (0.0139)^*^	0.0226 (0.0138)^*^	0.0329 (0.0163)^*^	0.0292 (0.0146)^*^	0.0466 (0.0186)^*^
Moderated mediation	0.0018 (0.0008)^*^	0.0017 (0.0009)^*^	0.0022 (0.0010)^*^	0.0016 (0.0008)^*^	0.0016 (0.0008)

*B, unstandardized coefficient; SE, standard error; PTS, post-traumatic stress; SOC, sense of coherence; PTG, post-traumatic growth*.

* *p < 0.05*.

PTG Overall and its subscales, except Life Orientation, moderated the association between Time 1 and 2 perceived severity, such that higher PTG at Time 2 is related to a more modest relationship between the two severity judgments of COVID (Table 6). Using Johnson-Neyman techniques, we identified 3.29 (Overall), 3.61 (Self), 3.79 (Interpersonal), and 3.03 (Spiritual) as the scores above which no significant association was found between Time 1 and 2 perceived severity, and they were slightly below the cutoff for substantial PTG.

**Table 6:** Effects of Time 1 perceived severity on Time 2 perceived severity at 16th, 50th, and 84th percentile of post-traumatic growth and its subscales (*N* = 327).

	**Effects [B(SE)] of Time 1 on Time 2 perceived severity at respective levels of PTG domains:**				
	**Overall**	**Self**	**Interpersonal**	**Spiritual**	**Life orientation**
16th percentile	0.5874 (0.0896)^***^	0.5632 (0.0910)^***^	0.6044 (0.0969)^***^	0.6609 (0.1010)^***^	0.5746 (0.1064)^***^
50th percentile	0.3723 (0.0816)^***^	0.3934 (0.0815)^***^	0.3710 (0.0838)^***^	0.3935 (0.0772)^***^	0.4313 (0.0777)^***^
84th percentile	0.2350 (0.1090)^*^	0.2721 (0.1119)^*^	0.2543 (0.1115)^*^	0.1261 (0.1232)	0.2880 (0.1111)^**^
Moderation effect	−0.2404 (0.0866)^**^	−0.1698 (0.0759)^*^	−0.1751 (0.0703)^*^	−0.2674 (0.0820)^**^	−0.1433 (0.0761)

*B, unstandardized coefficient; SE, standard error; PTG, post-traumatic growth*.

*Statistical significance of the effects was indicated by*

* *p < 0.05*;

** *p < 0.01*;

*** *p < 0.001*.

## Discussion

This study was conducted to investigate the PTG that emerged from the first COVID outbreak in Hong Kong in Spring 2020. The outbreak occurred shortly after intense social unrest, under a low level of trust to the public authority (Ni et al., 2020) and a highly divisive social fabric (Wong, 2020). Although the experience of SARS might have been conducive to high vigilance among the local public against the COVID pandemic (Kwok et al., 2020; Lau et al., 2020a), the public could be heavily stressed and traumatized. About one in four (28.4%) of our participants exhibited probable PTSD during the height of the first outbreak in March/April 2020. This estimate is higher than that of Ni et al. (2020) collected during the Anti-ELAB social movement in late-2019 and those from Lau et al. (2005) during SARS in May/June 2003. Yet, our estimate is comparable to those from studies conducted at the beginning of the COVID pandemic elsewhere (e.g., Fawaz and Samaha, 2020; González-Sanguino et al., 2020; Liu et al., 2020; Shevlin et al., 2020). Thus, the case in Hong Kong, even without a lockdown at the time of data collection, was just as traumatizing as those of elsewhere with more severe outbreaks.

The evidence on the direction and strength of the associations between PTS and PTG has been inconclusive (Dekel et al., 2011; Marziliano et al., 2020). In this study, we followed the transformational model of Tedeschi and Calhoun (2004) and included sense of coherence as a moderator modulating the association between PTS and PTG. As expected, our findings show that PTG was more likely to emerge in participants with high levels of both SOC and PTS. In other words, perceiving the outbreak as more severe and the emotional distress from the pandemic could be functional, as long as the individuals perceive themselves as capable of making sense of and managing the crisis. However, when coping resources are running low, the affective blunder may not entail growth at best or counterproductive at worst. With low SOC, stressful exposure to COVID was counterproductive for PTG in Self and Interpersonal domains. In fact, the importance of coping resources is corroborated by our adjacent finding that shows higher educational attainment, and higher income is facilitator of PTG. Hence, our findings underscore the importance of maintaining, or even building, coping resources—psychological and practical—during a stressful time for harvesting growth out of the difficult circumstances. We also tested and detected a sense of coherence as a factor leading to different directions of association between PTS and PTG.

Incomplete mediation was found with Overall PTG and Life orientation, meaning that some factors other than the coupling of PTS and SOC were contributing. Life orientation emphasized one's wish to change life priorities, which involves recognizing the status quo as less than desirable. This is unlike other subscales that emphasize the appreciation for the present—oneself, one's social circle, and spirituality. Admitting one's status quo as no longer beneficial may require a slightly different set of mental resources than SOC. For instance, having compassion to accept oneself for being vulnerable to nonoptimal situations may facilitate growth in Life orientation (Wong and Yeung, 2017). Another local study (Lau et al., 2020b) also found that self-compassion reduces the adverse impacts of perceived threats in the financial, social, work/academic, and family domains on mental health, rendering one more psychologically resilient in stressful times.

Compared to Ng (2020) where 47.4% of their sample in China reported PTG in at least one domain, relatively fewer of our participants experienced substantial PTG at Time 2. Ng (2020) remarked that the cut-off on PTG was stringent—on a scale from 0 to 5, the cut-off requires an average response of four. Yet, the responses of our sample clustered around the middle option (i.e., 2) with a reasonable spread. The difference between the Hong Kong and China estimates may be attributed to the differences in social contexts. Although a direct comparison of social cohesion with, Ng (2020) was not possible, missing a social fabric that encourages collective action and mourning may hinder PTG (Páez et al., 2007; First et al., 2018). Local polls have reported paramount dissatisfaction with the local authority on their handling of the pandemic (Cheung and Wong, 2020; Hong Kong Institute of Asia-Pacific Studies, 2020). Such dissatisfaction may have fueled the frustration among the public, and therefore their pessimism toward effective resolution of the pandemic. Furthermore, instead of a pervasive lockdown, Hong Kong remained largely open during the outbreaks, even though there was a record drop in year-to-year GDP (−8.9%) and quickly rising unemployment rates (Census and Statistics Department, 2020). The impact of pandemic on economy is likely to be unequal, with some industries, such as tourism, catering, and retail, being hit harder than others due to the social distancing policies. Anticipating severe but unequal pandemic aftermaths may enlarge the socio-economic divide and stifle collective growth. Echoing the call for examining the social determinants of health (Marmot and Wilkinson, 2005), future studies should identify the “social determinants of growth” for advancing a relational- or community-oriented account of PTG.

Although the temporal gap between the assessment of PTS and PTG was relatively short, this study has neatly captured experience of people at the peak of an outbreak and its subsequent palliation. For two reasons, we believed that there has been enough time for PTG to emerge among the Hong Kong general public. First, the citizens are not foreign to respiratory COVID as most adults have lived through the 2003 SARS epidemic. Second, news about a lethal viral outbreak has been looming in Hong Kong since the beginning of January 2020 (Chan and Jun, 2020). Thus, the city has been “on guard” well before the first local outbreak. Accordingly, the citizens reacted quickly with high perceived severity and prevalence of preventive measures, such as mask-wearing (Kwok et al., 2020; Lau et al., 2020a). The relatively high vigilance in the community could have triggered substantial cognitive processing and coping among the citizens since the beginning of 2020. In fact, Lau et al. (2006) have captured significant positive mental health impacts of SARS in the general public only 3 months after the first case. Hence, there should have been enough time for PTG related to the initial COVID outbreak to emerge in Hong Kong. However, we too agree that PTG is not necessarily static and may fluctuate with new perspectives of the stressful experience (Occhipinti et al., 2015; Stein et al., 2018). Hence, studies with a longer follow-up period are needed to ascertain how lasting the initial PTG will be, and the impacts of such PTG on coping with the long-term consequences of the COVID pandemic.

Lastly, our study has also found that participants with higher PTG, except Life orientation, tend to show a more modest association in severity judgments between the peak and the trough of the outbreak. In other words, PTG may foster adroit revision of risk perceptions according to the changing circumstances and reduce the unnecessary arousal, vigilance, and anxiety. To support the practical benefits of PTG, future studies may focus on capturing the behavioral implications of PTG, in addition to the cognitive and emotional implications.

## Practical Implications

Using Viktor Frankl's meaning triangle, Li (2020) highlighted “creativity, mobility, compassion, and resilience” as the key coping strategies among Hong Kong citizens during the Spring 2020 outbreak. Despite the ongoing distress, our findings show growth could be attained from adapting to the challenging “New Normal.” Low-cost and high-reach interventions using tele-counseling that foster awareness of signs of distress, appreciation of coping efforts, small blessings in daily lives, and attainment of meaning from changed routines have been proposed as good starting points (Holmes et al., 2020). Our findings underscore the salutary role of SOC. There has been evidence suggesting that mindfulness-based interventions may enhance SOC (Weissbecker et al., 2002; Ando et al., 2011). In fact, our findings may have offered an alternative perspective to understanding the effectiveness of mindfulness-based interventions, which is one through PTG. Referring to the salutogenesis model, Super et al. (2016) also put forward the behavioral and perceptual mechanisms for fostering SOC, with the first mechanism—empowerment—referring to strengthening the ability of people to identify the resources for coping (e.g., seeking help from trusted others), and the second mechanism—reflection—referring to being aware of own assumptions, values, and goals behind one's understanding of the stressful event. Thus, transparent and accessible pandemic- and support-related information and counseling for facilitating reflections toward one's suffering and coping with the pandemic could be useful for fostering SOC and therefore PTG.

## Limitations

The retention of participants from Time 1 was modest (372/761 = 48.9%). The large dropout was mainly due to the refusal of participants to leave their contact for a Time 2 survey. Non-random sampling may have also resulted in sampling bias that affects the generalizability of the results. Males, elderly persons, and individuals with low SES were under-represented. These individuals may be hit harder by the pandemic due to the precarity of their occupations, finances, and lower accessibility to formal and informal support. Hence, while our estimates of PTS could be an underestimation, and the rate of PTG could have been overestimated. We have also relied on only two time points to arrive at an indicator reflecting the flexibility of pandemic severity judgments, which has been taken as a proxy of PTG's effect on coping. Future studies may investigate how PTG accrued from the initial phase of a continuous stressor (e.g., the COVID-19 pandemic) affects trajectories of adaptation as the distressing context unfolds. Lastly, with respect to the sensitive socio-political landscape during the data collection period, we did not measure participants' exposure to and impacts from the social unrest. Future studies may examine personal and collective post-traumatic growth in politically polarized locations with more elaborate measurements of the impacts of political orientations and attitudes.

## Conclusion

The COVID pandemic has become one of the most severe pandemics since the 1918–1920 Spanish influenza. Our findings demonstrate that individuals with greater PTS and SOC reported more positive relationships between severity judgment at the outbreak peak and domains of PTG subsequently. Also, PTG was related to greater flexibility in severity judgments between the peak and the trough of the outbreak. These findings illustrate both the ingredients and the salutary impacts of PTG.

## Data Availability Statement

The raw data can be obtained upon request from the authors, without undue reservation.

## Author Contributions

All the authors contributed significantly to the conception, data collection, and writing up of the study. BL conducted the data analysis. All authors contributed to the article and approved the submitted version.

## Funding

The study is supported by Research Development Supporting Fund awarded to SN.

## Conflict of Interest

The authors declare that the research was conducted in the absence of any commercial or financial relationships that could be construed as a potential conflict of interest.

## Publisher's Note

All claims expressed in this article are solely those of the authors and do not necessarily represent those of their affiliated organizations, or those of the publisher, the editors and the reviewers. Any product that may be evaluated in this article, or claim that may be made by its manufacturer, is not guaranteed or endorsed by the publisher.
